# Computer-Aided Colon Polyp Detection on High Resolution Colonoscopy Using Transfer Learning Techniques

**DOI:** 10.3390/s21165315

**Published:** 2021-08-06

**Authors:** Chia-Pei Tang, Kai-Hong Chen, Tu-Liang Lin

**Affiliations:** 1 Division of Gastroenterology, Department of Internal Medicine, Dalin Tzu Chi Hospital, Buddhist Tzu Chi Medical Foundation, Chiayi 62247, Taiwan; franktg@hotmail.com; 2 School of Medicine, Tzu Chi University, Hualien City 97004, Taiwan; 3 Department of Management Information Systems, National Chiayi University, Chiayi 60054, Taiwan; kaihong@mis.ncyu.edu.tw

**Keywords:** object detection, medical information systems, colon polyp detection, colonoscopy, transfer learning

## Abstract

Colonoscopies reduce the incidence of colorectal cancer through early recognition and resecting of the colon polyps. However, the colon polyp miss detection rate is as high as 26% in conventional colonoscopy. The search for methods to decrease the polyp miss rate is nowadays a paramount task. A number of algorithms or systems have been developed to enhance polyp detection, but few are suitable for real-time detection or classification due to their limited computational ability. Recent studies indicate that the automated colon polyp detection system is developing at an astonishing speed. Real-time detection with classification is still a yet to be explored field. Newer image pattern recognition algorithms with convolutional neuro-network (CNN) transfer learning has shed light on this topic. We proposed a study using real-time colonoscopies with the CNN transfer learning approach. Several multi-class classifiers were trained and mAP ranged from 38% to 49%. Based on an Inception v2 model, a detector adopting a Faster R-CNN was trained. The mAP of the detector was 77%, which was an improvement of 35% compared to the same type of multi-class classifier. Therefore, our results indicated that the polyp detection model could attain a high accuracy, but the polyp type classification still leaves room for improvement.

## 1. Introduction

Colorectal cancer (CRC) is one of the leading causes of death worldwide. Colon polyps are proven to be a type of precancerous lesion. Early detection and elimination of this cancer precursor, the colon polyp, reduces the incidence of CRC [1]. Colon polyp (adenoma) detection and removal in colonoscopy decreases the mortality rate by 53%, as noted in a prospective study [2]. Colonoscopy is regarded as the primary screening tool and the gold standard by experts in the field for patients at risk of CRC [3].

The adenoma detection rate (ADR) is the quality performance indicator of an endoscopist. It is defined as the percentage of each colonoscopy examination that identifies more than one adenoma [4]. Corley et al. reported that each 1% increase of ADR was related to a 3% reduction in the incidence of CRC and a 5% decrease in the interval CRC-related fatality [5]. Kaminski et al. demonstrated similar results, finding that increasing the ADR to 24% can decrease the interval CRC mortality to 2.7% [6].

The primary goals of colonoscopies are to inspect the entire colon mucosa and locate any polyps. The two factors that prevent a thorough survey of the colon mucosa and increase the missed polyp rate are either mechanical or operator dependent. The mechanical factor includes poor bowel cleaning, with residual stool or debris, the colon lumen not being adequately distended and polyps being hidden behind the colon curves and folds. Polyps which have a tiny or flat morphology, have similar surrounding mucosa color or left colon polyps are easily missed, and contribute to the operator dependent factor [7]. The colon polyp miss rate is high in conventional colonoscopies and estimated to be 25% for all polyps and 26% for adenomas. In one study, nearly one quarter of polyps were missed in high resolution colonoscopies [7]. There is a need to overcome these factors to prevent missed polyps through development of the polyp recognition system. 

One of the challenges in decreasing the polyp miss rate is to recognize unseen polyps in the visual field [8]. This “inattentional blindness” phenomena occurs while the endoscopist completely misses polyps in their visual eye field because they are focusing on a different event and a new vision is not seen. Technologies to enhance the visual field include cap-assisted, extra-wide-angle, Retro View colonoscopy, and the water exchange (WE) method. Among the methods, WE provides superb bowel cleanliness and optimal platform for computer-aided detection (CAD) [9,10]. Studies show that an additional observer, either a gastroenterology trainee, fellow or endoscopy nurse, may improve the ADR up to 30% [11,12,13]. Real-time CAD serves as a second observer and has gained attention in recent years. It fills the gap to improve polyp detection and classification as it is low-cost, high sensitivity and consistent with immediate on-screen annotation. CAD will thus be able to replace human second observers in the near future. 

**Figure 1:**
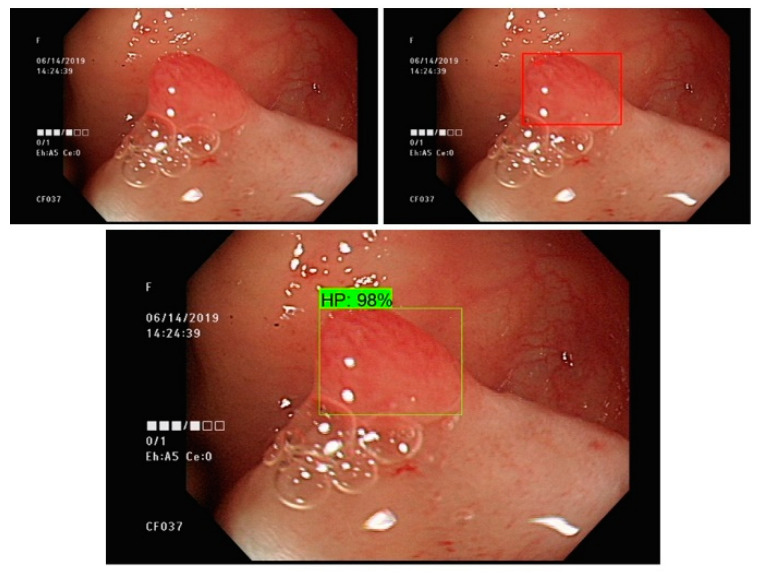
Results of polyp detection and classification system ((**upper left**): original colonoscopy, (**upper right**): annotated polyp, (**below**): CAD bounding box with classification).

We developed a real-time polyp detection and classification system based on different deep learning algorithms. Various types of non-neoplastic and neoplastic polyp static images with white light and NBI were used to establish an accurate detection and classification model. Our Convolutional Neuro Network (CNN) model was able to achieve low latency and was engineered to facilitate endoscopists in accurate colon polyp detection and classification, decreasing the missed polyps rate and preventing CRC (Figure 1).

## 2. Literature Review

### 2.1. Computer-Aided Colon Polyp Detection

The earliest development of computer-aided detection (CAD) dated back to the 1990s. Pixel-based features used intensities, thresholds and different segmentation approaches for pattern recognition on static images. In 2003, Karkanis et al. applied a color wavelet feature on static pictures and video frames validated on color colonoscopy videos with a sensitivity of 90% [14]. Before the era of deep CNN, the most common classifiers used for polyp recognition were the Supportive Vector Machine and K-nearest neighbor [15].

In recent years, the easy acquisition of large image databases has facilitated real-time polyp detection development. Fernandez et al. used an energy map for detecting polyps and determining their boundaries. They attained a 70.4% sensitivity and 72.4% specificity for polyp detection [16]. Urban et al. developed a polyp detection system using static images from screening colonoscopies. They achieved an accuracy rate of 96% for polyp detection [17]. Misawa et al. trained a CAD system that reached a high sensitivity of 90% but had a low specificity of 63.3% and an accuracy of 76.5% [18]. Klare et al. showed a CAD real-time system with a slightly enhanced ADR [19]. Wang et al. and Liu et al. further proved the efficacy of the CAD system in ADR improvements compared with colonoscopies conducted without CAD [20]. Until recently, most studies have been focused on polyp detection. All of the studies used CNN as their training and testing model platform. However, a CNN-related system for real-time polyp detection with classification has not yet been reported. 

There is evidence that deep CNN takes advantage of many sets of image layers, followed by pooling them in order to decrease data complexity in real-time colon polyp detection [21]. CNN incorporates constraints and gains deformation invariance through three concepts: local receptive field, shared weights and spatial subsampling. Among these, the shared weights decrease the number of parameters in the system and support generalization that makes a successful application within the different fields for image recognition [22]. Thus, CNN is the first choice for polyp detection system development. The other concern is the speed between the polyp recognition and the appearance of the bounding box, defined as latency. The latency is essential for any real-time procedure, and polyp detection is no exception. Tajbakhsh et al. reported their latency on polyp detection at 0.3 s with a sensitivity of 88% [17]. This latency is sufficient for human eyes to capture colon polyps with a bounding box. 

To better detect and classify colon polyps, a narrow band imaging (NBI) technique has been developed in recent years. NBI is an electronic chromoendoscopy which filters the traditional illuminating white light into blue and green colors, which are then absorbed by blood vessels, enhancing observation of the colon mucosa microstructure and vascularity. It is widely and routinely used in endoscopy suites in order to improve polyp detection and histologic prediction. Hewett et al. established a validation system to classify small polyps in hyperplastic and adenomatous using NBI colonoscopy. They attained an accuracy of 89%, sensitivity of 98% and 95% negative predictive value within real-time evaluation [23]. Deep neuro network (DDN)-CAD combined with an NBI system was developed to assist in more accurate polyp detection and classification. Chen et al. gained an identification rate of hyperplastic and neoplastic polyps with 96.3% sensitivity and 78.1% specificity. They found an outstanding intra-observer agreement between expert endoscopists, novice endoscopists and CAD. Among them, DNN-CAD had the best agreement in terms of polyp classification and shortest time required for polyp detection [24].

### 2.2. Convolutional Neural Networks

Recently, due to the automated feature selection characteristics, deep learning models attained great breakthroughs in computer vision [25,26,27,28]. Traditional computer vision algorithms greatly adopt manual engineered attributes, such as attributes from SURF (Speeded Up Robust Features) [29] and SIFT (Scale-Invariant Feature Transform) [30]. After the attributes were obtained, the obtained attributes were provided to the learning models in order to carry out recognition. The performance of the classical computer vision methods greatly relies on the quality of the obtained attributes. Nonetheless, attribute extraction itself is an intricate and burdensome job. While confronted with diverse questions or applications, re-designing the attributes is common and such a dispiriting job is normally named feature engineering. Feature engineering demands specialist domain knowledge and substantial traditional computer vision techniques are needed to manually re-engineered and re-choose image features for different applications. Currently, since the surge of deep learning, utilizing CNN can automatically attain feature extraction without requiring artificial feature engineering.

The deep learning approaches, such as CNN, have conquered traditional feature engineered models in many image recognition competitions, such as the ImageNet Large Scale Visual Recognition Challenge. Thus, employing CNNs in medical image recognition will aid in attaining greater precision than traditional artificial intelligence models.

**Figure 2:**
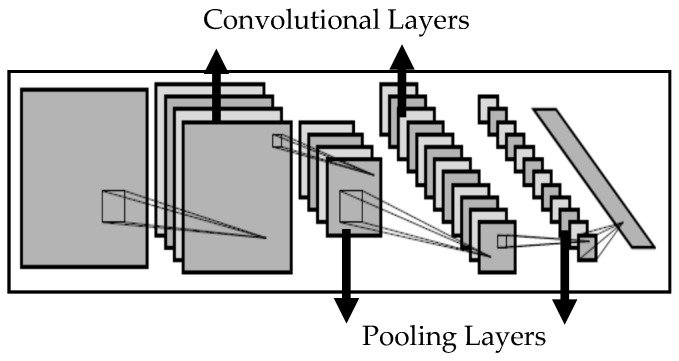
Schematic illustration of CNN [26].

In the fields of deep learning algorithms, CNN is no doubt the most successful approach. It has been proven to be able to out-perform humans in some colonoscopy recognition applications [27]. The expectation is that the artificial intelligence algorithm is able to precisely anticipate the annotations, regardless of colonoscopy deformation, rotation, translation or reduction. CNN utilizes convolution operations in order to process the colonoscopy bounding boxes and estimate the extent of similarity. Further, the pooling operation has been adopted in order to pick the largest number from the fixed-shape pooling window. The pooling operation may be seen as a colonoscopy compression approach and, after pooling, the entire number of dots may be greatly decreased. In short, the network layers of CNN could be broken down into two main types: the convolution operation type and the pooling operation type. Neurons were utilized within every operation to process weights and produce results. Figure 2 is a conceptual illustration of the convolutional neural network [26].

It can be said that the CNN is an artificial intelligence algorithm evolved from the neural system. The initial idea and essential architecture behind the artificial neural network (ANN) is to imitate the neuron learning process in neurobiology that is able to determine weights through a list using straightforward and rapid computation [31]. Akin to actual neurons, the character of artificial neuron in the ANN is to add the weighted numbers and then convert the sum to an output number, according to different activation functions. There are many activation functions, such as the hyperbolic tangent or sigmoid transformation. The computation of an ANN may be parallel distributed and computed, and an ANN model may be obtained through the given sample itself, so that the data analysis is not bound by the assumption of the sample selection [32].

### 2.3. Object Detection Using CNN

Although the CNN may be utilized to conduct polyp detection and generate the annotations, CNN alone is not able to recognize the region of the selected targets. There may be more than one kind of polyp and tumor in the colonoscopy images, so it could be crucial to utilize object detection approaches in order to identify the location and the range of various targets from the colonoscopy and conduct multi-category recognition on the colonoscopy images. The most direct technique to achieve this would be to utilize the sliding window idea. This is the idea that fixed-size windows are adopted to stretch through the full colonoscopy individually and that colonoscopy frames can be introduced to the CNNs to resolve classifications. Since the size of the target is uncertain, it is crucial to adopt distinctive window sizes in order to conduct recognition. Nonetheless, the sliding window concept is an expensive computational technique that needs to examine entire colonoscopy over several cycles. Due to the sliding window conception, the entire computation drains substantial computational resources and is only acceptable when the frames per second requirement is low. To counter this, the use R-CNN is proposed [33]. Instead of going through over the entire colonoscopy, R-CNN pre-selects roughly 2000 potential regions and then forecasts the possible regions individually.

Faster R-CNN is an enhanced variant of R-CNN. Rather than pre-selecting region proposals, Faster R-CNN chooses region proposals simply from a feature map computed using the CNN [34]. Faster R-CNN utilizes another CNN named RPN (Region Proposal Network). The contribution of the RPN is to take the features directly from the feature map, which is obtained from the front CNN, and derive bounding boxes and the probabilities that the bounding boxes consist of the targets. After RPN, the top probable bounding boxes can be procured. Even if the coordinates of these bounding boxes are not exact, the coordinates can be adjusted using RoI (Region of Interest) Pooling. After RoI pooling, every region is rapidly annotated and the best bounding box coordinates are discovered.

In Faster R-CNN, a RoI-wise subnetwork is adopted in order to conduct the detection, but is unnaturally implanted. Thus, R-FCN, a fully convolutional network, is introduced [35]. R-FCN utilizes a position-sensitive score to consolidate the translation variant characteristics within the design. Because of the direct scheme of the fully convolutional network, R-FCN runs faster than Faster R-CNN.

In spite of achieving good accuracy, the inherited two-stage form in Faster R-CNN causes the identification speed to be considered inferior in comparison with one-stage detection methods. An alternate method, the SSD (Single Shot MultiBox Detector), which is a one phase approach that does not re-sample the bounding box proposals, was presented [36]. SSD has some advantages in terms of the running time.

## 3. Materials and Methods

### 3.1. Dataset

This is a retrospective study of diseased and healthy individuals who underwent colonoscopy exams in a secondary teaching hospital. The ethics committee of Tzu-Chi General Hospital, Da-Lin branch in Taiwan approved this study. Colonoscopy exam dates and personal medical records were gathered only if the electronic medical report indicated that the patient had polyps. Pictures of each colonoscopy image set that contains polyps, white light or NBI, were collected in JPEG format. Detection is mainly affected by the resolution of the colonoscopy image. Although the JPEG is encoded by lossy compression, the detection rate is not significantly affected. Each polyp was correlated with the respective pathology report and classified as tubular adenoma, hyperplastic polyp, tubulovillous adenoma, sessile serrated adenoma or adenocarcinoma. All polyp images were labeled by an experienced endoscopist. Our predictive model development was based on 70% of a set of random partitioning histology-validated polyps as the initial training set. The other 30% of polyps were used for model testing and validation. Our dataset incorporated 2843 polyp images in total. The classes and the size of the annotated samples used in this work is presented in Table 1.

In this study, a LabelImg image marking tool was adopted to mark the polyps. LabelImg allows the user to drag a rectangle containing a polyp, and to store the center coordinates and length and width of the rectangles using the xml format. A common technique utilized in object detection to increase the detection rate is data augmentation. Simple methods, such as zooming, cropping, flipping, and rotating can improve the accuracy of the model. In this study, flipping and zooming is included in the object detection algorithm and the number of samples of small polyps is increased by zooming, so it is suggested that zooming improves the model’s ability to detect small polyps.

The categories of our dataset and their annotated samples include HP, hyperplastic polyp; HPNBI, hyperplastic polyp in narrow-band imaging; SSA, sessile serrated adenoma; SSANBI, sessile serrated adenoma in narrow-band imaging; TA, tubular adenoma; TANBI, tubular adenoma in narrow-band imaging; TUMOR, adenocarcinoma; TUMORNBI, adenocarcinoma in narrow-band imaging; TVA, tubulovillous adenoma; and TVANBI, tubulovillous adenoma in narrow-band imaging.

### 3.2. Problem Formulation

The polyp recognition task can be divided into the training phase and testing phase. During the training stage, n records of colonoscopy images data, the region of the polyp and polyp types, are provided as the training set, and the training set can be formatted as following.

(1) $$T\;=\{\left({\mathrm{Img}}_{1},\{({\mathrm{bb}}_{11},l_{11}),\left({\mathrm{bb}}_{12},l_{12}\right),\ldots ,\left({\mathrm{bb}}_{1m_{1}},l_{1m_{2}}\right)\}\right),\;({\mathrm{Img}}_{2},\{({\mathrm{bb}}_{21},l_{21}),\left({\mathrm{bb}}_{22},l_{22}\right),\ldots ,\left({\mathrm{bb}}_{2m_{2}},l_{2m_{2}}\right)\}),\ldots ,\;({\mathrm{Img}}_{i},\{({\mathrm{bb}}_{i1},l_{i1}),\left({\mathrm{bb}}_{i2},l_{i2}\right),\ldots ,\left({\mathrm{bb}}_{{\mathrm{im}}_{i}},l_{{\mathrm{im}}_{i}}\right)\}),\ldots ,\;\left({\mathrm{Img}}_{n},\{({\mathrm{bb}}_{n1},l_{n1}),\left({\mathrm{bb}}_{n2},l_{n2}\right),\ldots ,\left({\mathrm{bb}}_{{\mathrm{nm}}_{n}},l_{{\mathrm{nm}}_{n}}\right)\})\right\}$$

where Img_i_ is the ith colonoscopy image, ${\mathrm{bb}}_{{\mathrm{im}}_{i}}$ is the m_i_th polyp region that consists of the coordinates of the polyp region, and $l_{{\mathrm{im}}_{i}}$ is the polyp type of the m_i_th polyp region within the ith colonoscopy image. When given a polyp image as an input, the finished training model can generate the predicted polyp regions and polyp types. The mathematical equation can be represented as the follows.

(2) $$\left\{\left({\mathrm{bb}}_{j1},{\;l}_{j1}\right),\left({\mathrm{bb}}_{j2},{\;l}_{j2}\right),\ldots \left({\mathrm{bb}}_{{\mathrm{jm}}_{j}},{\;l}_{{\mathrm{jm}}_{j}}\right)\right\}=F\left({\mathrm{Img}}_{j}\right)$$

When given a polyp image Img_j_ as an input, the prepared model $F\left({\mathrm{Img}}_{j}\right)$ should generate a few polyp regions that indicate the coordinates of the region and the types of the polyp. The examination system will decide whether the generated polyp region and polyp and tumor types agree with the given polyp region and polyp types.

**Figure 3:**
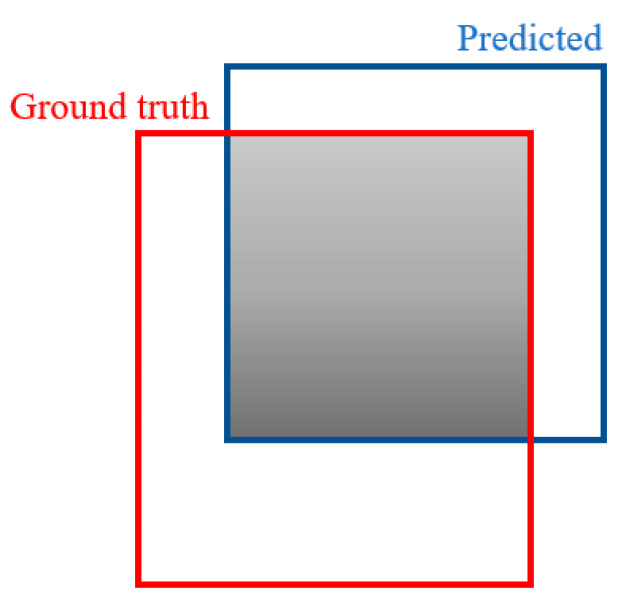
Intersection of union.

When identifying the polyp and tumor using the detection system, the polyp region should be spotted. In Figure 3, a mathematical approach to analyze the annotated ground-truth polyp region and the detected polyp region is the IoU (Intersection over Union) [37], as demonstrated. The IoU was adopted as a metric to assess the precision of the recognized polyp region with the corresponding polyp within an appropriate colonoscopy. While a polyp region is computed from the result, the IoU was derived utilizing the following Equation (3).

(3) $$\mathrm{IoU}\left(A,B\right)=\left|\frac{A\cap B}{A\cup B}\right|$$

where A denotes the ground-truth polyp region, and B denotes the predicted polyp region. Consequently, to gauge the IoU of any size and shape, the following regions are required.

1. ground-truth region of the polyp
2. detected region of the polyp

To work on the estimation of the IoU, instead of irregular shapes, rectangular boxes or bounding boxes will be utilized to conduct the IoU computation for comparison.

### 3.3. Methods

To distinguish polyps and tumors, polyp detection technology is introduced. While the CNN can conduct polyp recognition when the input polyp is provided and the CNN can figure out what type the polyp or tumor is, only the classification, and not the location, can be recognized within the CNN detection computation.

In any case, simply accomplishing polyp and tumor class recognition is not enough. It is important to spot the position and size of various disease regions within the colonoscopy. Consequently, the object detection approach is embraced for multi-class and regional selection.

Numerous object detection approaches are introduced, such as R-CNN [33], Fast R-CNN [38], Faster R-CNN [34] and Mask R-CNN [39]. Regardless of the use of R-CNN or Fast R-CNN, the pre-selection of the region proposals through selective search [40] is still crucial, and a selective search is a better computation approach compared with the sliding window method. Consequently, in this work, we utilized Faster R-CNN, R-FCN and SSD to build the kernel of the polyp and tumor recognition framework. Faster R-CNN omits the region proposal selection and selects region proposals from the CNN’s feature map in a straightforward fashion. Faster R-CNN utilizes a convolution neural network named the Region Proposal Network. The feature map from the principal CNN was provided to the Region Proposal Network. Accordingly, the rectangular polyp regions and the probabilities of the polyp types could be obtained at the last phase, so that the top probable polyp regions and polyp types might be selected.

**Figure 4:**
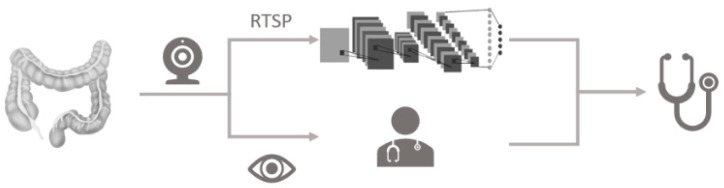
The concept of the polyp identification framework.

The idea of the polyp identification framework is presented in Figure 4. There are 3 stages as follows:

1. Employ the video capture card to capture the video colonoscopy stream from the endoscopy device.
2. Utilize the Real Time Streaming Protocol (RTSP) to import the signal from the colonoscopy device and conduct real-time polyp detection with deep learning framework.
3. Adopt the deep learning framework to forecast the type of the polyp and region. Choose the polyp region and generate the coordinates for further verification.

In this research, we use mAP (mean Average Precision) to measure the different object detection approaches. The mAP computation works by having an AP computation for all classes and then calculating the average. In short, the AP is the mean precision for one class and the mAP is the mean precision for all classes. The AP was derived using the following Equation (4).

(4) $$\mathrm{AP}=\frac{1}{11}\underset{r\in \left\{0,0.1,\ldots ,1\right\}}{\sum }P_{\mathrm{interp}}\left(r\right)\;\mathrm{where}\;P_{\mathrm{interp}}\left(r\right)=\underset{\tilde{r}:\tilde{r}\geq r}{\mathrm{maxp}}\left(\tilde{r}\right)$$

Normally, in the mAP computation, the Precision Recall curve will be plotted at the beginning. In Equation (3), P is the precision and r is the recall. The computation determines the largest precision where recall is larger than a fixed limit. We set the limit to 0.1 for a better outcome in this research. Typically, the limit is fixed to 0.5. Because the limit is fixed to 0.1, the gradual increase is also 0.1. There are a total of 11 recall numbers ranging from 0 to 1. In this way, the sum is partitioned by 11 in Equation (4).

**Figure 5:**
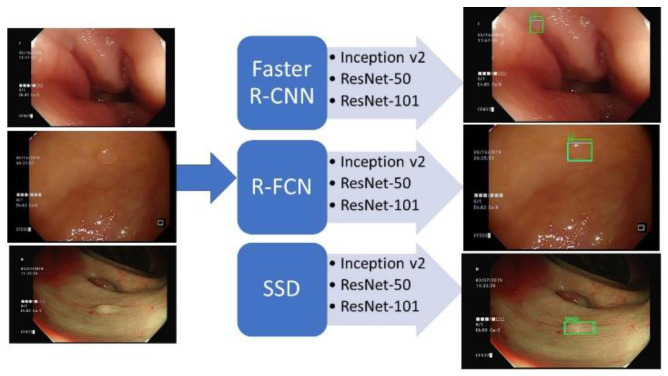
Polyps detection system.

Figure 5 demonstrates the design of the polyp detection system. The system contains 3 recognition algorithms—R-FCN, Faster R-CNN and SSD—and 3 network structures—ResNet-50, ResNet-101 and Inception v2. There are 9 combinations that could be used to detect the polyps. Given the initial annotated polyp regions, the models were trained to identify the polyps and tumors. When given polyp images, recordings and online video streams, the trained model can produce the recognized regions and polyp’s classes.

## 4. Experimental Results

### 4.1. Model Training

**Figure 6:**
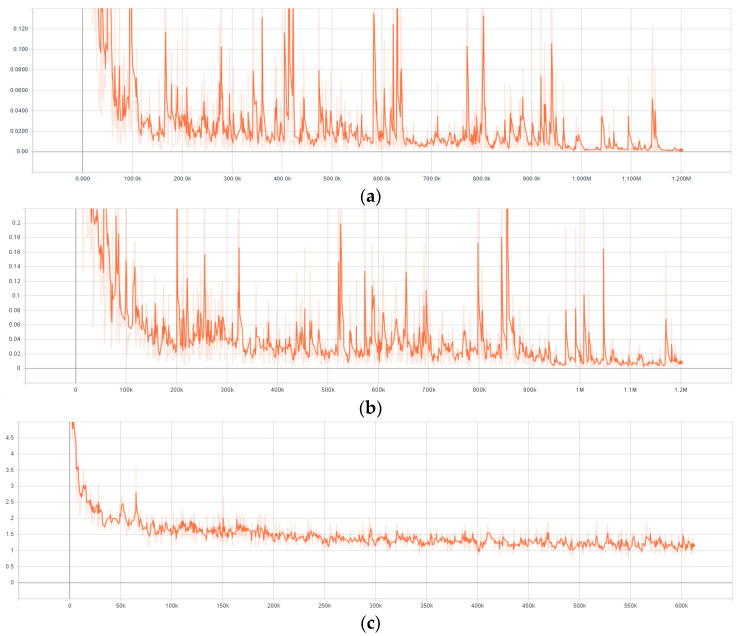
Loss function of (**a**) Faster R-CNN with ResNet_101 (**b**) R-FCN with ResNet_101 (**c**) SSD with ResNet_101.

The presented models, Faster R-CNN, R-FCN and SSD, have been prepared and tested using GPU of NVidia GeForce GTX 2080ti on a Processor PC with CPU of Intel Core i5 8500 3.0 GHz. Figure 6 demonstrates the end-to-end loss function (Losses_Total Loss) of the 3 models using ResNet_101 during the training process. It can be seen from Figure 6 that the more extensive the training process, the smaller the total loss. Although the loss functions did not converge smoothly, the values of the loss function decreased gradually. The fluctuation of the loss function is due to the setting of the learning rates and because of the decrease trends. Thus, the training can be deemed to be effective. Hence, the trend shows that the prepared model is valid. The loss function of the test set fluctuates around the converged value.

### 4.2. Detection Results

**Figure 7:**
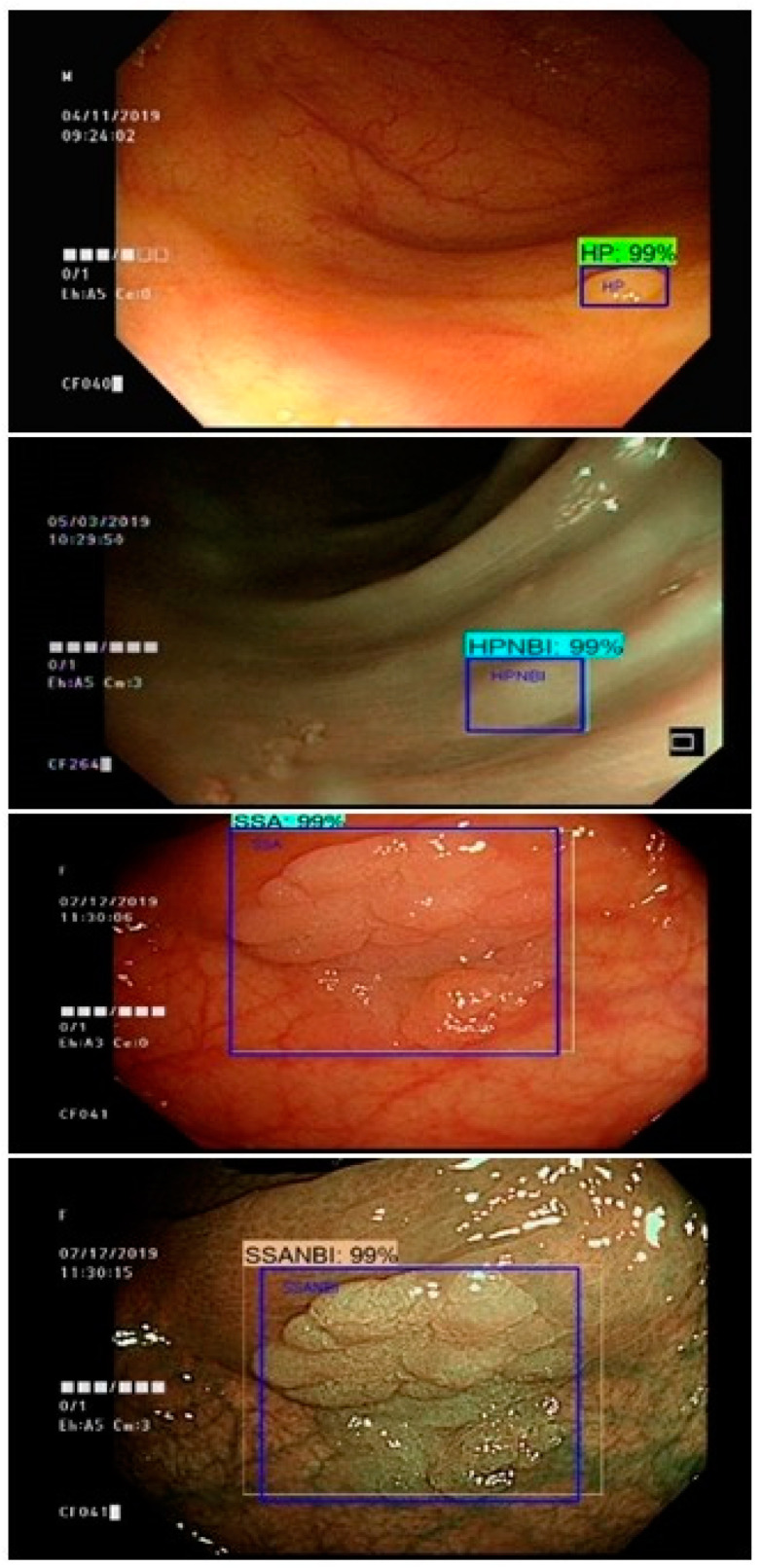
Detection outcomes of HP and SSA with and without NBI (blue box: expert annotated region, other: machine prediction).

While preparing the neural systems, repeat learning and memory are fundamental so that the ultimate trained model will be able to recognize the polyps in the real-time colonoscopy while images, recordings and online video streams are being fed to the models. The frame per second (FPS) rate of the algorithms ranged from 6 to 22. Figure 7 demonstrates the ultimate detection outcomes and the spotted region within the colonoscopy image. In Figure 7, colonoscopy images that contain the polyp regions are annotated by specialists and the machine detection outcomes are listed with and without NBI.

Table 2 demonstrates the outcomes of mAP computation for Faster R-CNN, R-FCN and SSD. The network structures adopted in this work are Inception v2, ResNet50, ResNet101. The outcomes were generated using the Google TensorFlow. The R-FCN with the ResNet101 network structure performs best. By the large, there ought to be room for advancement. It is interesting to note that colonoscopies with NBI perform well in R-FCN, no matter whether they are within TA, HP or TVA. The performance of Faster R-CNN in TA, HP or TVA demonstrates no significant improvement when the NBI is applied to the colonoscopy.

When using Faster R-CNN, the mean AP did not differ substantially across the three network structures, Inception v2, ResNet-50 and ResNet-101, so the Inception v2 is remains the optimal choice due to its better frame rate. Compared with Faster R-CNN and SSD, R-FCN performs surprising well in most of the cases in NBI. Thus, the model performance can be improved further when the colonoscopy switches to NBI and chooses R-FCNN as the detection method.

When we compared TANBI, HPNBI and TUMORNBI, TANBI and TUMORNBI could be detected more precisely. Olympus has developed an NBI and the NBI adds a set of filters to the original white light colonoscope light source, with a wavelength of 400 nn~700 nn which allows for blue light with a wavelength of 415 nn and green light with a wavelength of 540 nn to pass. The growth environment of all neoplastic polyps is mostly distributed around blood vessels, because blood vessels can provide the nutrients needed for polyps. Therefore, TA and TUMOR display more distinguishable characteristics in NBI and the result is consistent with previous studies.

**Figure 8:**
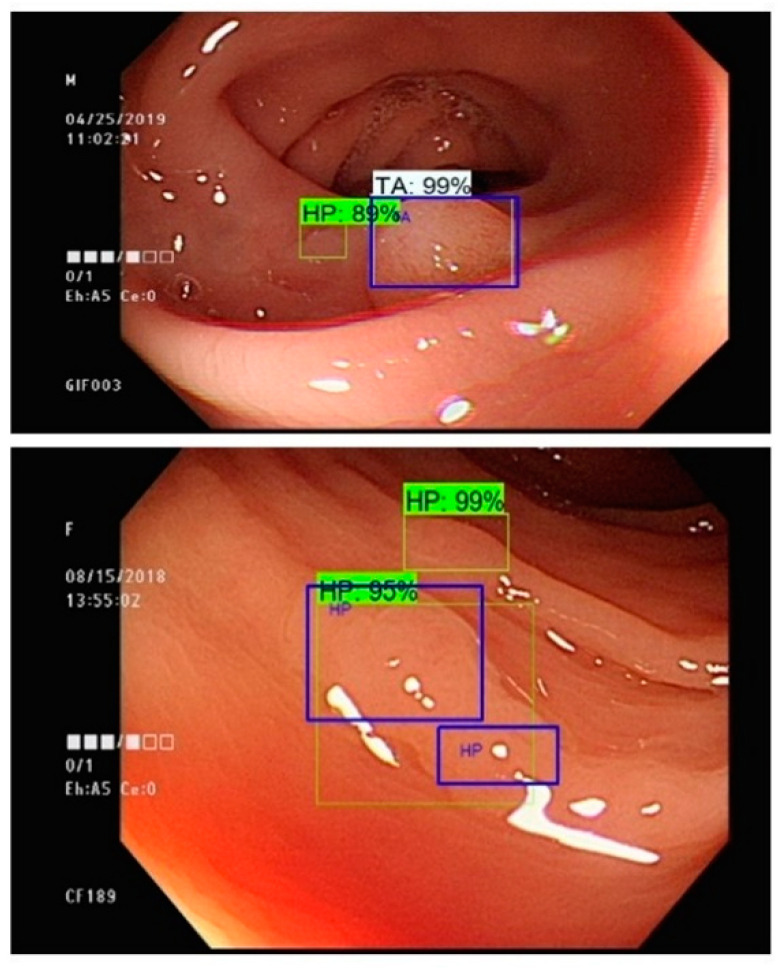
The detection outcomes using Faster R-CNN with Inception v2 network structure (blue box: expert annotated region, other: machine prediction).

Multiple polyp regions are identified in Figure 8. Surprisingly, an additional suspicious region is detected by the prediction system without any human mediation. We believe this discovery can advance the precision of the polyp and tumor detection system. In this manner, a creative reinforcement learning process is inspired. The dataset can be approximately separated into two datasets: training and testing. Both training and testing data are comprised of annotated colonoscopies with the polyp regions and polyp classes. The training data then will be utilized in order to prepare the model. The prepared model will be tested using the testing dataset. Testing outcomes are generated from the testing process. The testing outcomes will then be given to the specialist for further verification. Any contentious cases within the testing outcomes will be further re-inspected and tested by the specialists and the adjusted examples will be incorporated into the training dataset. After several reinforcement cycles, the annotated mistakes can be adjusted, and the accuracy of the model can be increased further. The discordance between machine and human can be greatly reduced through the introduction of a reinforcement model learning process.

**Figure 9:**
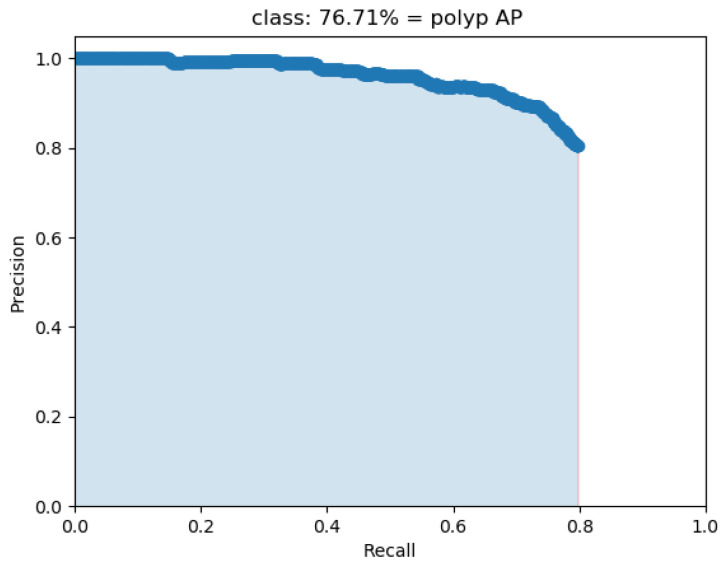
The PR curve for the binary polyp identification system.

Since the mAP of the multi-class classifer based on Faster R-CNN with Inception v2 in Table 2 was only 42%, we conducted another experiment. The poor outcome might be due to the 10 classes that have been used within our model. Therefore, a binary classifier for polyp detection is trained using the same dataset. The binary classifier adopted the same Faster R-CNN with the Inception v2 model. Compared to the multi-class classifier, the mAP of binary classifier is 77%, which is about a 35% improvement. The PR curve of the binary classifier is presented as Figure 9.

## 5. Conclusions

In order to reduce the incidence of CRC through the recognition and early resecting of colon polyps, we have created an automatic detection system that can detect polyps and tumors in real-time colonoscopies. This system assists the endoscopist to detect polyps in their early stages, decreasing the missed polyp rate, preventing CRC and decreasing CRC mortality. As a result, the health cost will be diminished. Faster R-CNN, R-FCN and SSD are adopted to develop the identification system. The R-FCN has the highest mAP among all models. We found the detection system could be improved further by adopting the introduced flow of reinforcement model development. Any controversial cases obtained from the testing outcomes can be further re-examined and confirmed by the endoscopy expert in the reinforcement cycle and ultimate altered cases can be fed again into the dataset. After a few fortification cycles, numerous polyp annotation errors can be adjusted and the model precision can be further improved. The disagreement between machines and humans can be solved with the introduction of fortification flow. Several multi-class classifiers were trained and the mAP ranged from 38% to 49%. A binary classifier which adopted Faster R-CNN with the Inception v2 model was trained. The mAP of the binary classifier was 77%, which was about 35% improved compared with the multi-class classifier. Therefore, these outcomes indicate that the polyp detection model could attain a high accuracy, but the polyp type classification still leave space for improvement.

Currently, the polyp detection dataset had 2483 polyp images. In spite of the fact that the recognition outcome is satisfactory, if the polyp database can be expanded, the recognition rate may be improved and better outcomes attained. The variety of polyps and tumors should be expanded, as a diverse dataset would be valuable in the future.

## Figures and Tables

**Table 1:** List of categories included in our dataset and their annotated samples.

Class	Number of Linages	Number of Annotated Samples ^a^
HP	340	407
HPNBI	111	155
SSA	249	274
SSANBI	49	59
TA	1121	1202
TANBI	252	269
TUMOR	40	50
TUMORNBI	19	21
TVA	256	333
TVANBI	46	53
Total	2483	2823

^a.^ Number of annotated samples after data augmentation.

**Table 2:** The mAP outcomes of Faster R-CNN, R-FCN and SSD.

Meta-Architecture					
	Faster R-CNN			R-FCN	SSD
Class/Feature Extractor	Inception v2	ResNet-50	ResNet-101	ResNet-101	ResNet-101
TA	0.68	0.60	0.67	0.64	0.53
TANBI	0.69	0.69	0.67	0.74	0.51
HP	0.48	0.39	0.50	0.48	0.29
HPNBI	0.25	0.33	0.50	0.51	0.29
TVA	0.36	0.35	0.26	0.31	0.25
TVANBI	0.26	0.14	0.12	0.42	0.51
SSA	0.36	0.40	0.44	0.38	0.38
SSANBI	0.15	0.20	0.23	0.28	0.23
TUMOR	0.31	0.35	0.31	0.46	0.44
TUMORNBI	0.67	0.67	0.67	0.67	0.67
Total mean AP	0.42	0.41	0.42	0.49	0.38

## Data Availability

Not applicable.
